# Carbon Monoxide Partially Mediates Protective Effect of Resveratrol Against UVB-Induced Oxidative Stress in Human Keratinocytes

**DOI:** 10.3390/antiox8100432

**Published:** 2019-10-01

**Authors:** Janice N. Averilla, Jisun Oh, Jong-Sang Kim

**Affiliations:** 1 School of Food Science and Biotechnology (BK21 Plus), Kyungpook National University, Daegu 41566, Korea; averillajanice@gmail.com; 2 Institute of Agricultural Science and Technology, Kyungpook National University, Daegu 41566, Korea; j.oh@knu.ac.kr

**Keywords:** resveratrol, heme oxygenase, carbon monoxide, mitochondria, UVB irradiation, keratinocytes

## Abstract

Based on the antioxidative effect of resveratrol (RES) in mitigating reactive oxygen species (ROS) production through the induction of nuclear factor-erythroid 2-related factor-2 (Nrf2)/heme oxigenase-1 (HO-1) signaling pathway, we investigated whether the protective activity of RES against ROS-mediated cytotoxicity is mediated by intracellular carbon monoxide (CO), a product of HO-1 activity, in ultraviolet B (UVB)-irradiated human keratinocyte HaCaT cells. The cells were exposed to UVB radiation following treatment with RES and/or CO-releasing molecule-2 (CORM-2). RES and/or CORM-2 upregulated HO-1 protein expression, accompanied by a gradual reduction of UVB-induced intracellular ROS levels. CORM-2 reduced intracellular ROS in the presence of tin protoporphyrin IX, an HO-1 inhibitor, indicating that the cytoprotection observed was mediated by intracellular CO and not by HO-1 itself. Moreover, CORM-2 decreased RES-stimulated mitochondrial quantity and respiration and increased the cytosolic protein expressions of radical-scavenging superoxide dismutases, SOD1 and SOD2. Taken together, our observations suggest that RES and intracellular CO act independently, at least partly, in attenuating cellular oxidative stress by promoting antioxidant enzyme expressions and inhibiting mitochondrial respiration in UVB-exposed keratinocytes.

## 1. Introduction

The human skin represents high vulnerability to external hazards, such as ultraviolet radiation (UV) from the sun, air pollutants, cigarette smoke, and other factors [1] and thus undergoes dynamic self-renewal and orchestrated repair processes [2,3,4]. At normal physiological conditions, normal metabolic processes concomitantly generate oxidants, more commonly known as the electrophilic reactive oxygen species (ROS), which are closely regulated by the elaborate antioxidant network to maintain cellular redox homeostasis [5]. Majority of the intracellular ROS originates from the electron transport chain as a part of normal energy-generating mechanisms of the mitochondria which ultimately produces adenosine triphosphate (ATP) biosynthesis [6,7,8].

Exposure to ultraviolet B (UVB) interrupts the internal redox homeostasis, which consequently results in dysregulation of cellular processes [9]. ROS are localized in the mitochondria, thereby potentially compromising the fundamental mitochondrial functionality [10]. Substantial exposure to UV radiation stimulates ROS production by initiating the transformation of molecular oxygen to unstable free radicals such as superoxide anion (O_2_•^−^), hydrogen peroxide (H_2_O_2_), and hydroxyl radical (OH•^−^) [11,12]. Although these chemical species are short-lived, their accumulation imposes potential skin damage via inflammatory cascades which culminate in inflammation and aging [13].

Our recent study has demonstrated that resveratrol (RES) exerts a protective effect by regulating UVB-induced ROS production in HaCaT cells via heme oxigenase-1 (HO-1) signaling [14]. In another study, resveratrol (RES) exemplified nuclear factor-erythroid 2-related factor-2 (Nrf2)-activating capacity and thus promotes transcription of antioxidant enzymes including heme oxygenase-1 (HO-1) [15], which closely controls the cellular redox balance [16]. The activation of Nrf2 and its downstream-regulated genes, including HO-1, superoxide dismutase 1 (SOD1), and superoxide dismutase 2 (SOD2), is a major contributor in RES-mediated cellular antioxidant mechanism [17,18]. The remarkable function of RES as a modulator of mitochondrial processes by modulating the mitochondrial respiratory activity in colon cancer cells has been formerly reported [19]. Although there have been numerous proposed mechanisms of RES-mediated biological effects at oxidative conditions, molecular events focusing on the HO-1/monoxide (CO) signaling pathway and its interplay with the mitochondria have not been explored to our knowledge.

In general, HO-1 is upregulated to confront oxidative stress [20] and catalyzes the conversion of heme into equimolar amounts of iron, CO, and biliverdin-Ixα, which is enzymatically reduced to bilirubin-Ixα [21,22]. CO is an odorless diatomic molecule, which has been considered earlier as a toxic air pollutant because it inhibits the function of the cytochrome c oxidase in the mitochondrial respiratory chain [23,24]. In humans, heme metabolism generates about 86% of endogenous CO, which enables apparent signaling functions in cellular processes [25,26]. CO has a substantially stronger interaction with heme compared to oxygen, leading to limited oxygen availability for oxidative phosphorylation [27]. Emerging evidence showing the role of CO as a gasotransmitter challenge the previous label of CO as merely a ubiquitous toxic gas [26]. For example, CO was found to modulate cytochrome c oxidase activity to limit oxidative stress in vitro and in vivo [28,29]. Additionally, controlled CO level was found to regulate cellular response against stress-inducing stimuli such as UV radiation [30,31].

Considering that RES protects HaCaT cells via HO-1 regulation and that the biological effects of HO-1 could be chiefly attributable to its enzymatic activity which liberates carbon monoxide (CO) and biliverdin [32,33], we hypothesized that RES potentially regulates cellular adaptive response via intracellular CO production in human skin (Figure S1). CO-releasing molecule-2 (CORM-2) employed in this study as a source of regulated quantities of CO emulates the activity of endogenous CO [34]. Moreover, its co-treatment with RES introduces how controlled levels of intracellular CO influence the protective function of RES against UVB-induced oxidative stress. Therefore, the current work was undertaken to investigate the potential role of intracellular CO in maintaining cellular homeostasis by upregulating the stress-inducible antioxidant enzymes to stimulate cellular detoxification and ultimately maintain redox balance in human skin cells.

## 2. Materials and Methods

### 2.1. Chemicals

Resveratrol (Purity ≥ 99%) and CORM-2 were purchased from Sigma-Aldrich (St. Louis, MO, USA). Both compounds were dissolved in dimethyl sulfoxide (DMSO) to achieve the desired stock solution concentrations. All other chemicals were procured from Sigma-Aldrich (St. Louis, MO, USA), unless otherwise specified, and employed without further purification.

### 2.2. Cell Culture

HaCaT cells, the most widely-used model of human keratinocytes, were received from Korean Cell Line Bank (Seoul, Korea) and were grown in monolayers in Dulbecco’s modified Eagle’s medium (DMEM) supplemented with 10% fetal bovine serum (FBS) and antibiotics (100 units/mL of penicillin and 100 μg/mL of streptomycin) in 10-cm transparent culture dish (SPL Life Sciences Co., Ltd., Gyeonggi-do, Korea) at 37 °C in an incubator supplied with 5% CO_2_. Cells were transferred every three to four days onto new culture dish, placing 1 × 10^5^ cells per dish at each tranfer.

### 2.3. Cell Viability

Cells (5 × 10^3^ cells/well) were seeded onto a 96-well transparent plate (SPL Life Sciences Co., Ltd., Gyeonggi-do, Korea), incubated overnight to reach approximately 80% confluency, and treated with various concentrations (0–200 µM) of CORM-2 in 0.5% FBS DMEM for 24 h. Separate wells were treated with 30 μM RES and/or 10 μM tin protoporphyrin IX (SnPP, HO-1 inhibitor). Cytotoxicity was evaluated using the Cell Counting Kit-8 (CCK-8; Dojindo Laboratories, Kumamoto, Japan) according to the manufacturer’s instructions. Absorbance was detected at 450 nm using an absorbance microplate reader (Sunrise^TM^, Tecan Grp. Ltd., Männedorf, Switzerland). Cell viability is reported as the percentage relative to the control (%). Since samples were dissolved in DMSO to prepare stock solutions, all assays in this experiment were performed using DMSO as a control treatment.

To test the cytoprotection against UVB- or H_2_O_2_-induced damage, cells were treated with the resulting non-toxic concentration of CORM-2 from the viability test above (100 µM), together with 30 µM RES and/or 10 µM SnPP. Six hours after sample treatment, cells were washed twice and suspended in phosphate-buffered saline (PBS), exposed to 50 μM H_2_O_2_ or irradiated with UVB (25 mJ/cm^2^, 290–320 nm, Spectrolinker XL-1000 UV Crosslinker, Spectronics Corp., Westbury, NY, USA), and allowed to recover for 24 h while maintained in 0.5% FBS in DMEM at 37 °C in an incubator supplied with 5% CO_2_. The intensity of UVB radiation was used consistently throughout this study. Finally, CCK-8 assay was performed shortly after the allotted recovery time and absorbance was detected at 450 nm. All determinations were conducted in three replicates and repeated at least twice.

### 2.4. Measurement of UVB-Induced Intracellular ROS Production

HaCaT cells were seeded (5 × 10^3^ cells/well) onto an opaque black 96-well plate (SPL Life Sciences Co., Ltd., Gyeonggi-do, Korea), incubated overnight, starved for 4 h in 0.5% FBS in DMEM, and treated with 30 μM RES, 10 μM SnPP, and/or 100 μM CORM-2 for 6 h. Conditioned media were transferred onto a new transparent 96-well plate and stored in the incubator. Cells were washed with phosphate-buffered saline (PBS) twice, exposed to UVB, and allowed to recover for 6 h in 0.5% FBS in DMEM. Intracellular ROS production was evaluated in accordance with a previously reported method with modifications [35]. Briefly, cells were incubated with 30 μM 2′,7′-dichlorofluorescein diacetate (H_2_DCFDA) for 30 min in the dark, followed by washing with PBS. Afterward, cells were reincubated in the conditioned media for another hour, and then fluorescence was measured using a fluorescence microplate reader (Infinite 200, Tecan, Grodig, Austria) at excitation and emission wavelengths of 485 and 535 nm, respectively. Results were calculated relative to the respective control cells and reported as percentage relative fluorescence unit (% RFU). All determinations were conducted in three replicates and repeated at least twice.

### 2.5. Time-Course Analysis of H_2_O_2_-Induced Intracellular ROS Generation

HaCaT cells were seeded (5 × 10^3^ cells/well) onto an opaque black 96-well plate (SPL Life Sciences Co., Ltd., Gyeonggi-do, Korea), incubated overnight, starved for 4 h in DMEM containing 0.5% FBS, and treated with 30 μM RES, 10 μM SnPP, and/or 100 μM CORM-2 for 6 h. Conditioned media were transferred into a clean 96-well plate and stored in the incubator. Cells were then washed with PBS, exposed to 50 µM H_2_O_2_ for 30 min, and allowed to recover for 1 h or 6 h in 0.5% FBS in DMEM. Intracellular ROS production was evaluated in accordance with the method described above. Results were calculated relative to the respective control cells and reported as percentage relative fluorescence unit (% RFU). All determinations were conducted in triplicates and repeated at least twice.

### 2.6. Measurement of Mitochondrial DNA Quantity by Quantitative Real-Time PCR

HaCaT cells (1 × 10^6^) were seeded in a 10-cm transparent culture dish (SPL Life Sciences Co., Ltd., Gyeonggi-do, Korea), treated with samples, irradiated with UVB, and then allowed to recover for 12 h. Afterward, cells were trypsinized and collected for DNA quantification. The total cellular DNA extraction was performed by using QIAamp^®^ DNA mini kit (Qiagen GmbH, Hilden, Germany) according to the manufacturer’s recommended procedure for cell cultures and was quantified by spectrophotometry (BioSpectrometer^®^ Basic, Eppendorf, Hamburg, Germany). The concentration of the DNA content obtained (A260/280 and A260/230) were expressed in ng/µL. Furthermore, quantitative real-time PCR (qRT-PCR) using LightCycler^®^ 96 qPCR System (Roche, Basel, Switzerland) in conjunction with LightCycler^®^ 480 Multiwell white plates (96-well) (Roche, Basel, Switzerland) was employed to measure the mitochondrial DNA (mtDNA) content in the samples. In brief, the mitochondrial copy number was obtained by calculating the relative proportion of mitochondrial DNA copy numbers (primers: forward 5′-CACCCAAGAACAGGGTTTGT-3′, reverse 5′-TGGCCATGGGTATGTTGTTA-3′; Bioneer, Daejeon, South Korea) to two nuclear reference DNA (primers: forward 5′-TGCTGTCTCCATGTTTGATGTATCT-3′, reverse 5′-TCTCTGCTCCCCACCTCTAAGT-3′; Bioneer). All qRT-PCR determinations were carried out in triplicates using protein content of 100 ng/well. All determinations were conducted in three replicates and repeated at least twice.

### 2.7. Measurement of Mitochondrial Oxygen Consumption Rate (OCR)

To evaluate real-time mitochondrial respiration, the oxygen consumption rate (OCR) in live HaCaT cells was monitored using Seahorse XFp Extracellular Flux Analyzer (Agilent Technologies, Inc., Santa Clara, CA, USA) accompanied by Agilent Seahorse Mito Stress Test kit (Agilent Technologies, Inc., Santa Clara, CA, USA). Cells (5 × 10^3^) were seeded in XFp cell culture microplates, treated with samples for 6 h, exposed to UVB, allowed to recover for 12 h. In addition, the sensor cartridge was simultaneously incubated in a 37 °C non-CO_2_ incubator to hydrate the sensor. Immediately after recovery time elapsed, basal respiration was measured and was followed by sequential injection of mitochondrial agents loaded in specific injection ports such as oligomycin (complex V inhibitor) for measuring ATP-linked and proton-leak OCR, cyanide-*p*-trifluoromethoxyphenyl-hydrazone (FCCP, respiratory uncoupler) for measuring maximal respiratory capacity, and rotenone/antimycin A (inhibitors of complex I and III) for measuring the non-mitochondrial respiration.

At least an hour prior to the assay, treatment media were discarded and cells were incubated in 180 µL assay medium (XF base medium, 10 mM glucose, 1 mM pyruvate, and 2 mM L-glutamine, pH 7.40) at 37 °C in a non-CO_2_ incubator. The analyzer was calibrated initially, and the microplates were subjected to Mito Stress Test. The OCR was recorded after three cycles of measurement and was normalized to protein content determined for each well by Bradford assay (Bio-Rad Protein Assay, Bio-Rad, Hercules, CA, USA) at the end of each OCR determination. Results were represented graphically and expressed as pmol/min/µg protein. Respiration linked to the key mitochondrial bioenergetic parameters was quantified and reported as relative basal respiration, ATP-linked respiration, proton leak, maximal respiration, spare respiration capacity, and non-mitochondrial respiration. All determinations were conducted in three replicates and repeated at least twice.

### 2.8. Preparation of Cytosolic Extract

HaCaT cells (1 × 10^6^) seeded in 10-cm dish (SPL Life Sciences Co., Ltd., Gyeonggi-do, Korea) and incubated for 24 h were treated with CORM-2 and/or RES for 6 h, irradiated with UVB, and then allowed to recover for 12 h. Afterward, cells were trypsinized (Trypsin-EDTA solution, Welgene, Gyeongsan, Korea), and collected for protein extraction. Cytoplasmic proteins were fractionated by applying NE-PER^®^ Nuclear and Cytoplasmic Extraction Reagents (NER and CER, Thermo Scientific/Pierce Biotechnology, Rockford, IL, USA) according to the manufacturer’s instructions. Briefly, harvested cells were lysed using CER I and II and then centrifuged at 10,000× *g* for 30 min at 4 °C. The resulting supernatant was considered as the cytoplasmic fraction and stored at −20 °C. Protein content was determined in both fractions using Bradford assay (Bio-Rad, Hercules, CA, USA) as indicated by the manufacturer. Lastly, lysates were combined with sample loading buffer (40 μg/15 μL buffer) containing 62.5 mM tris-HCl pH 6.8, 2% sodium dodecyl sulfate (SDS), 0.1% bromophenol blue, 5% β-mercaptoethanol, and 20% glycerol, and then rapidly heated at 95 °C for 5 min. The extracts were labeled and stored at 0–20 °C prior to Western blot analysis.

### 2.9. Protein Electrophoresis and Western Blotting

The cytoplasmic protein samples prepared above were separated through sodium dodecyl sulfate-polyacrylamide gel electrophoresis (SDS-PAGE) and then transferred to a polyvinylidene fluoride (PVDF) membrane (Merck Millipore Corp., Billerica, MA, USA) at 100 V for 60 min. The membrane was blocked in 1% bovine serum albumin (BSA)-Tris-buffered saline/Tween20 (TBS/T, 20 mM Tris-HCl, pH 7.4, 150 mM NaCl, 0.1% Tween 20) for at least 2 h and the desired proteins were incubated overnight with the appropriate antibodies anti-SOD1, anti-SOD2, anti-β-actin (Santa Cruz Biotechnology, Inc., Dallas, TX, USA), and anti-HO-1 (Abcam, Cambridge, UK). Secondary antibodies anti-rabbit IgG and anti-mouse IgG conjugated to horseradish peroxidase (HRP) (Santa Cruz Biotechnology, Inc., Dallas, TX, USA) for 4 h. β-Actin serves as the internal loading control. Finally, membranes were washed thrice with TBS/T for 30 min and the protein bands were visualized using Super Signal^TM^ West Pico Chemiluminescent Substrate (Thermo Scientific, Waltham, MA, USA) and ImageQuant LAS 4000 Mini (GE Healthcare Life Sciences, Little Chalfont, UK). Images were analyzed densitometrically using Image Studio Lite version 5.2 (LI-COR Corp., Lincoln, NE, USA). All determinations were conducted in three replicates and repeated at least twice. 

### 2.10. Statistical Analysis

Results were analyzed by one-way analysis of variance (ANOVA) using the SPSS Statistics 22 software (SPSS Inc., Chicago, IL, USA). Statistical significance among mean values was determined by Duncan’s multiple range tests at *p* < 0.05. Statistical differences among values are represented by different alphabetical letters. Values sharing common letters show no significant differences.

## 3. Results

### 3.1. Intracellular CO Protects HaCaT Cells from UVB- and H_2_O_2_-Induced Oxidative Damage Possibly via HO-1 Regulation

**Figure 1:**
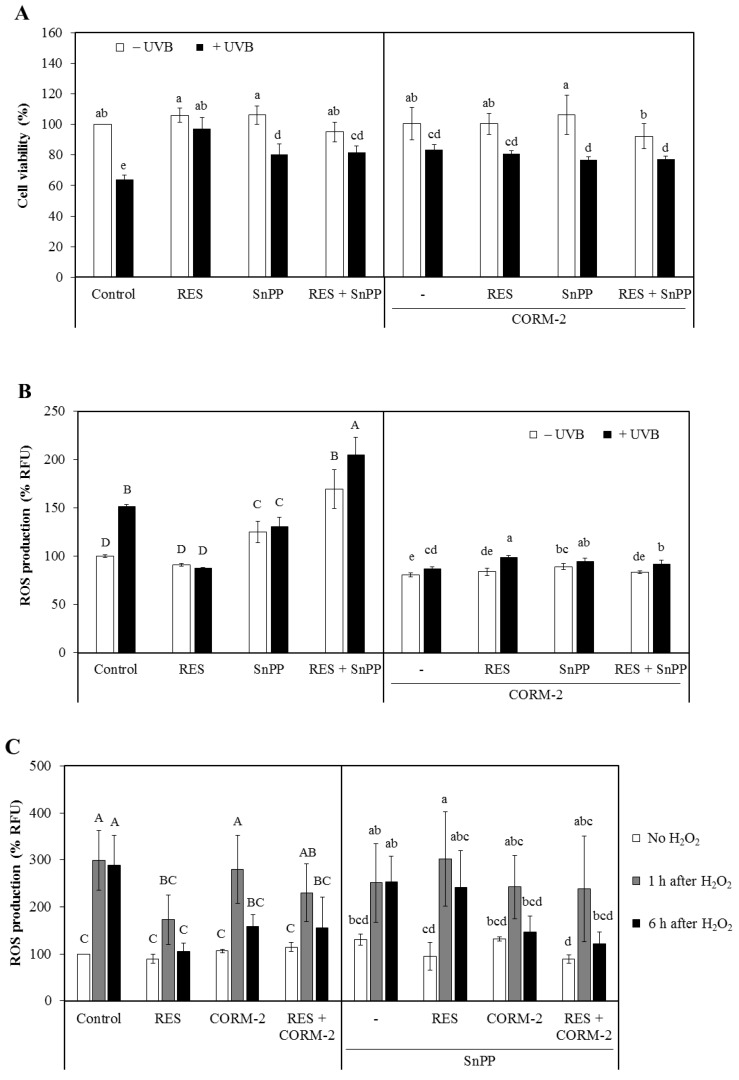

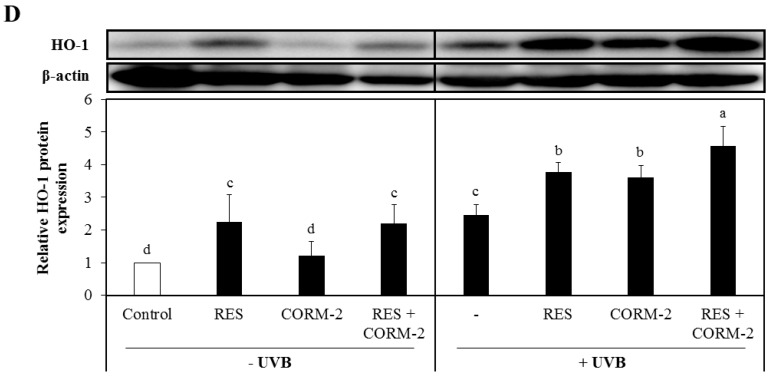
Cytoprotective effect of resveratrol (RES) and CO-releasing molecule-2 (CORM-2) against oxidative damage in HaCaT cells. (**A**) ultraviolet B (UVB)-exposed HaCaT cells pre-treated with RES and/or CORM-2. CORM-2 and RES increased the viability of cells exposed to UVB. Cells were pre-treated with RES, CORM-2, and/or SnPP, exposed to UVB, and then subjected to CK-8 cytotoxicity assay. (**B**) Combinatorial treatment of RES, CORM-2, and SnPP significantly ameliorated UVB-induced intracellular reactive oxygen species (ROS) production in HaCaT cells. Co-treatment of CORM-2 and SnPP abolished the intracellular ROS production in UVB-irradiated cells. Cells were pre-treated with RES, CORM-2, and/or SnPP, exposed to UVB, and then intracellular ROS was measured by H_2_DCFDA assay. (**C**) Time-course analysis of intracellular ROS generation in H_2_O_2_-exposed cells pre-incubated with CORM-2, RES, and/or SnPP showed a gradual reduction in intracellular ROS with treatment of RES or CORM-2. CORM-2 reduced intracellular ROS after heme oxigenase-1 (HO-1) inhibition. Cells were pre-treated with RES, CORM-2, and/or SnPP for 6 h, exposed to 50 µM H_2_O_2_, and then intracellular ROS was measured by H_2_DCFDA assay. (**D**) Treatment with RES and/or CORM-2 significantly increased HO-1 protein expression in UVB-irradiated HaCaT cells. Cells were pre-treated with RES, CORM-2, and/or SnPP for 6 h, exposed to UVB, and then harvested for Western blotting. Results represent mean ± SD (*n* = 3). Bars not sharing common letters indicate significant differences (*p <* 0.05), as evaluated using one-way ANOVA followed by the Duncan multiple-range test. Upper case letters in bar graphs indicate that the data were compared among each other. Similarly, lower case letters were compared among each other. Control, DMSO; RES, resveratrol (30 µM); CORM-2, CO-releasing molecule-2 (100 µM); SnPP, tin protoporphyrin IX (10 µM); % RFU, % relative fluorescence unit.

Initially, HaCaT cells were exposed to various concentrations of CORM-2 for 24 h. CORM-2 treatment exhibited no toxicity at concentrations equal to or lower than 100 μM in HaCaT cells (Figure S2). Thus, 100 μM CORM-2 was applied in the succeeding experiments to test the cytoprotective effect of intracellular CO against UVB- or H_2_O_2_-induced cytotoxicity. Additionally, non-cytotoxic concentrations of RES (30 μM) and SnPP (10 μM) were employed throughout the study. Afterward, the cytoprotective effect of RES and CORM-2 against UVB-induced cytotoxicity was evaluated (Figure 1A). Cells were incubated with RES, CORM-2, and/or SnPP for 24 h, exposed to UVB, and then were given a 12-h recovery period. Co-treatment with SnPP was performed to simultaneously examine the effect of CORM-2 when HO-1 activity is suppressed. Results show that RES and CORM-2 significantly increased cell viability in cells exposed to UVB, suggesting that RES and CORM-2 stimulate cell resistance to UVB-induced oxidative stress (*p* < 0.05). To evaluate whether CORM-2 could ameliorate intracellular ROS generation caused by increased levels of H_2_O_2_ after UVB exposure [36,37], H_2_O_2_ was applied exogenously on cells pre-incubated with RES, CORM-2, and SnPP. Co-treatment of CORM-2 and SnPP enhanced cell viability against H_2_O_2_-induced toxicity, suggesting that intracellular CO undertakes the protective function of HO-1 activity (Figure S3).

The damaging consequences of UVB in cells have been greatly correlated to its high energy and capacity to initiate or accelerate the formation of ROS, resulting in unregulated oxidative stress [38]. Hence, quantifying the UVB-induced intracellular ROS production to evaluate the potential contribution of intracellular CO in maintaining cellular redox homeostasis under oxidative conditions is essential. To investigate the protective roles of RES and CORM-2 against UVB-induced oxidative stress, HaCaT cells were incubated with RES and/or CORM-2 for 6 h and then exposed to UVB with a 6-h recovery period. The UVB-induced intracellular ROS production was quantified using H_2_DCFDA, a fluorescent probe which allows quantification of intracellular H_2_O_2_ [39]. UVB treatment significantly increased intracellular ROS production and was aggravated by SnPP (*p* < 0.05). Treatment with SnPP before UVB exposure abolished resistance to UVB-induced oxidative stress even in cells pre-treated with RES, indicative of the key role of RES-induced HO-1 in ameliorating oxidative damage. On the other hand, UVB-induced intracellular ROS production was ameliorated with co-treatment of RES, CORM-2, and SnPP after a 6-h period recovery (Figure 1B). Remarkably, co-treatment of CORM-2 and SnPP mitigated the UVB-induced intracellular ROS production, suggesting that CORM-2 substitutes the cytoprotective effect of HO-1 activity (*p* < 0.05).

Considering that the cytotoxicity of UVB involves unregulated formation of ROS and UVB-induced damage is correlated with excessive H_2_O_2_ generation [40,41], the extent of UVB-induced oxidative stress was examined through a 6-h time-course analysis of intracellular ROS production with H_2_O_2_ exposure (Figure 1C). H_2_O_2_ treatment significantly increased intracellular ROS levels and pre-treatment with SnPP aggravated its effect, suggesting that HO-1 is closely associated with the removal of ROS. H_2_O_2_-induced intracellular ROS production was significantly reduced with RES pre-treatment. However, co-treatment with SnPP inhibited its protective effect from oxidative stress, confirming that RES-mediated reduction in intracellular ROS level is achieved through HO-1 activity. Fascinatingly, co-treatment of CORM-2 with RES and/or SnPP inhibited H_2_O_2_-induced intracellular ROS, implying that the RES-mediated cytoprotection presumably inhibited by SnPP could be restored upon CORM-2 treatment. Moreover, the intracellular ROS formation was initially increased following treatment with H_2_O_2_ for 1 h but was reduced gradually over a 6-h recovery period in cells pre-treated with RES and CORM-2, suggestive of a progressive adaptive process that cells undergo to maintain homeostasis. Although further tests are required to directly examine the effect of HO-1-derived intracellular CO and directly determine its ability to substitute the function of HO-1, these findings could provide preliminary information on the ability of intracellular CO to inhibit the progression of intracellular ROS generation in a time-dependent fashion.

Given that accumulation of intracellular ROS signals the internal cellular antioxidant response system [42] and that the current data indicate the important role of HO-1 in alleviating UVB-induced oxidative stress, the protein expression of the key antioxidant enzyme involved in ROS modulation, HO-1, was measured by Western blot analysis. HaCaT cells were treated with RES and/or CORM-2 for 6 h, irradiated with UVB, and then allowed to recover for 12 h. UVB irradiation significantly upregulated the protein expression of HO-1 in HaCaT cells treated with RES and/or CORM-2, evidently demonstrating that the intrinsic protective mechanism of cells against oxidative stress is stimulated by RES and/or CORM-2 (Figure 1D).

### 3.2. Intracellular CO Alleviates UVB-Induced Oxidative Damage by Modulating Mitochondrial Functionality

**Figure 2:**
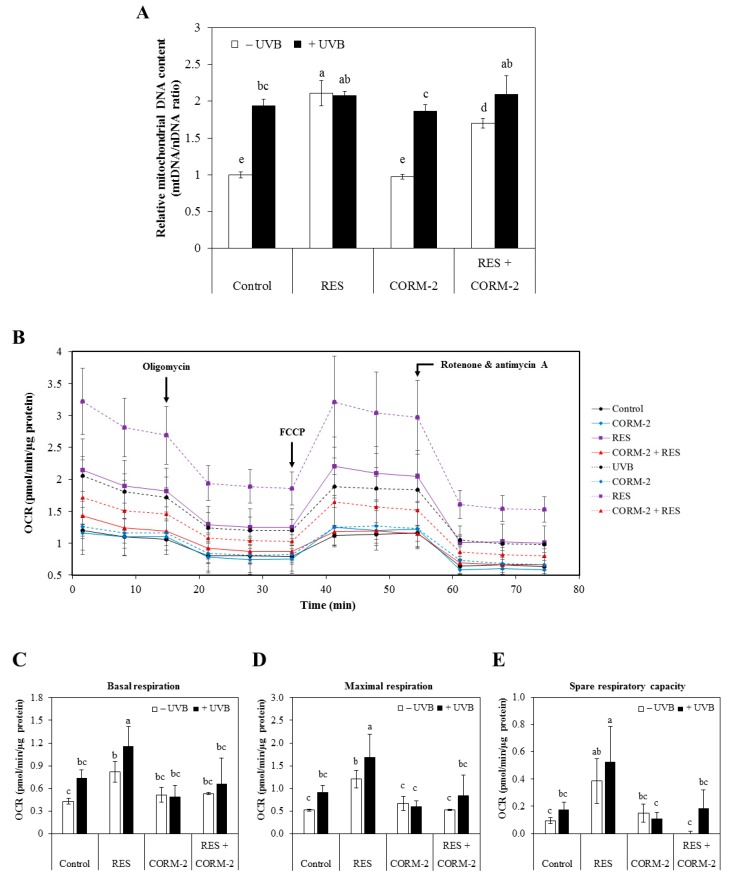
Modulation of mitochondrial DNA quantification and respiration by RES and/or CORM-2. (**A**) RES, but not CORM-2, increased the ratio of mitochondrial DNA to nuclear DNA, indicative of increased mitochondrial biogenesis. UVB also induced mitochondrial biogenesis and was maintained in cells pre-treated with RES and CORM-2, suggesting that mitochondrial number is optimized to prevent oxidative damage. (**B**) In general, mitochondrial respiration was increased by UVB and was substantially increased further in cells pre-treated with RES. CORM-2 mildly inhibited mitochondrial respiration to the control level. Mitochondrial OCR was measured by sequentially applying different mitochondrial inhibitors using Seahorse Mito Stress Test. Mitochondrial OCR related to key parameters of mitochondrial function were derived such as (**C**) basal respiration, (**D**) maximal respiration, and (**E**) spare respiratory capacity. RES increases OCR linked to key parameters of respiration while CORM-2 treatment showed mild inhibition. Cells were pre-treated with RES and/or CORM-2 and then exposed to UVB. Results represent mean ± SD (*n* = 3). Bars not sharing common letters indicate significant differences (*p <* 0.05), as evaluated using one-way ANOVA followed by Duncan’s multiple-range test. Upper case letters in bar graphs indicate that the data were compared among each other. Similarly, lower case letters were compared among each other. Control, DMSO; RES, resveratrol (30 µM); CORM-2, CO-releasing molecule-2 (100 µM).

With previous studies reporting the interference of intracellular CO with mitochondrial function via the electron transport chain [23], the cytoprotective role of intracellular CO through modulation of mitochondrial function at oxidative conditions was examined. Considering that RES improves mitochondrial function and biogenesis in various models of aging and metabolic diseases and that UVB leads to mitochondrial dysfunction in HaCaT cells [42,43], we examined whether intracellular CO promotes resistance against UVB-induced cytotoxicity by improving mitochondrial function. Thus, we measured mitochondrial biogenesis after independent or combinatorial treatment of RES and CORM-2 for 6 h in the presence or absence of UVB irradiation with a 12-h recovery period. Results showed that RES dramatically stimulated mitochondrial biogenesis in the absence of UVB exposure while CORM-2 retained the level similar to the control (*p* < 0.05) (Figure 2A). In the literature, RES promotes mitochondrial biogenesis through stimulation of Sirtuin 1, which leads to deacetylation and activation of the key regulator of mitochondrial production, called peroxisome proliferator gamma coactivator 1 alpha [43]. However, co-treatment with CORM-2 seemed to slightly inhibit this effect (*p* < 0.05). Similarly, UVB exposure significantly elevated mitochondrial number as represented by mtDNA content which was maintained even with independent or combinatorial treatment of the respective samples (*p* < 0.05). Although our data clearly indicated that CORM-2 showed mild inhibition of mitochondrial biogenesis regardless of UVB treatment, our findings are consistent with the previous study which exhibited the increase of mitochondrial biogenesis by RES [43]. Moreover, in contrast to our initial prediction that CO may increase mitochondrial biogenesis in the same manner as RES, our data rather suggest a more complex mechanism of RES action in terms of mitochondrial biogenesis not limited to RES-induced HO-1-mediated CO production.

Taking our results above into consideration, we further speculated that CO regulates mitochondrial functionality rather than mitochondrial biogenesis. At present, one of the most widely used strategies in evaluating mitochondrial function in living cell populations is through quantification of mitochondrial respiration, which can best be represented by mitochondrial OCR [44]. Thus, we examined mitochondrial OCR in the presence or absence of UVB irradiation in sample-treated HaCaT cells by Seahorse Mito Stress Test. Cells were treated with RES and/or CORM-2, irradiated with UVB, allowed to recover for 12 h, and then subjected to live cell Mito Stress Test. The OCR linked to the key parameters of mitochondrial respiration such as basal respiration, ATP-linked respiration, proton leak, maximal respiration, spare respiratory capacity, and non-mitochondrial respiration was calculated based on the results obtained by applying respective mitochondrial inhibitors. The mitochondrial inhibitors employed during the course of the assay selectively suppressed target enzymes that participate in the electron transport chain for ATP synthesis and thus allow estimation of mitochondrial functionality under stressed conditions [45].

Results clearly demonstrated that the mitochondrial respiration was increased after UVB treatment and was increased further in cells pre-treated with RES. In general, CORM-2 mildly suppressed mitochondrial respiration to values similar to the control level, and thus showing that a controlled inhibition of mitochondrial respiration was non-cytotoxic (Figure 2B). The basal respiration in cells pre-treated with RES, with or without UVB irradiation, was significantly higher compared to the non-irradiated control cells (*p* < 0.05) (Figure 2C). UVB treatment amplifies the effect of RES on basal respiration, as demonstrated by the further increase in mitochondrial oxidative metabolism with UVB treatment. On the other hand, CORM-2 treatment showed no effect on the basal respiration with or without UVB. In fact, its co-treatment with RES mildly inhibited OCR in the presence or absence of UVB.

Immediately following the measurement of basal respiration, the mitochondrial pharmacological agents were introduced to the respiring cells sequentially in order to estimate the OCR parameters directly influenced by ATP production, proton leak, and reserve capacities. Results revealed that RES promotes an increase in maximal respiration and reserve capacities (Figure 2D–E). Impairment of the mitochondrial respiratory capacity ultimately leads to diminished mitochondrial health, an important factor which influences health and disease development [46,47]. Collectively, our results support previous evidence of beneficial effects from the regulation of mitochondrial OCR by RES [48], indicative of improved mitochondrial function and thereby alleviates UVB-induced oxidative stress. Although CORM-2 consistently showed no effect on mitochondrial OCR with or without UVB when applied alone, its co-treatment with RES showed considerable inhibitory effects, confirming the potential function of intracellular CO in modulating cellular processes. According to literature, CO inhibits mitochondrial respiration through inhibition of the electron transport chain [23,24,26,28,29,30]. However, this was not observed when CORM-2 was applied alone but was detected upon co-treatment with RES and UVB. We suppose that the concentration of CORM-2 used in this experiment was not sufficient to induce any effect on mitochondrial respiration when applied singly, but significant enough to elicit an inhibitory response with RES and UVB application. Most importantly, CORM-2 possibly limits electron transport and oxygen consumption in the mitochondrial respiratory chain by inhibiting cytochrome c oxidase [29], but only to a non-toxic level, which is similar to that of our control treatment.

### 3.3. Intracellular CO Increases Protein Expression of the Antioxidant Enzymes SOD1 and SOD2

**Figure 3:**
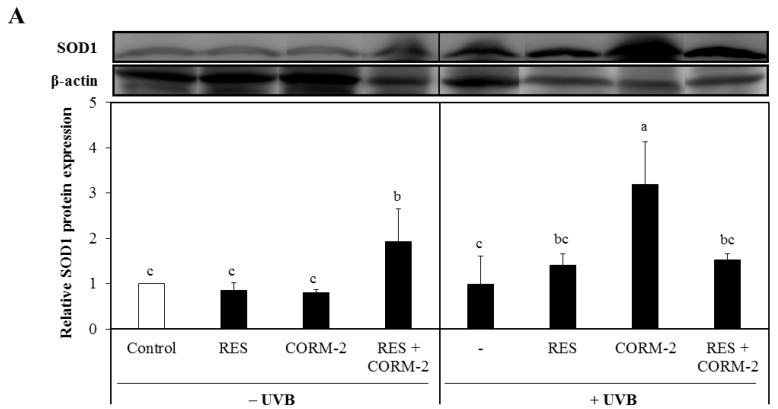

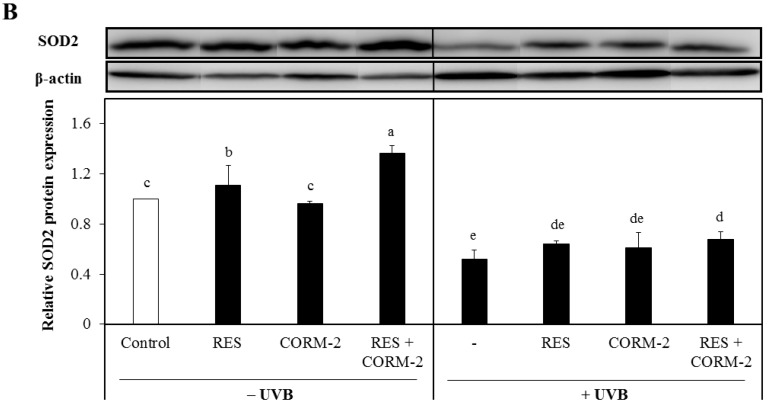
Effect of RES and/or CORM-2 on cytosolic protein expression levels of superoxide dismutases UVB-irradiated HaCaT cells. (**A**) SOD1 protein expression level of UVB-irradiated cells pre-treated with RES and/or CORM-2. With UVB, CORM-2 significantly induced SOD1 protein expression. (**B**) Cytosolic SOD2 protein expression of cells pre-treated with CORM-2. Without UVB, CORM-2 and RES induced SOD2 protein expression. After UVB treatment, a substantial reduction in cytosolic SOD2 was observed. HaCaT cells were pre-treated with RES and CORM-2, alone or in combination, and then exposed to UVB. Bars not sharing common letters indicate significant differences (*p <* 0.05), as evaluated using one-way ANOVA followed by Duncan’s multiple-range test. Upper case letters in bar graphs indicate that the data were compared among each other. Similarly, lower case letters were compared among each other. Control, DMSO; RES, Resveratrol (30 µM); CORM-2, CO-releasing molecule-2 (100 µM).

Considering that suppression of mitochondrial respiration stimulates mitochondrial-derived ROS formation [29], which is intensely regulated by the function of SOD [49,50], we examined the cytosolic protein expression levels of SOD1 and SOD2 in cells treated with RES and/or CORM-2 to assess the contribution of CO and ROS signaling in alleviating UVB-induced oxidative stress. Although both enzymes are abundantly expressed in the cytosol, activation of SOD2 causes its migration into the mitochondria [51,52]. Results demonstrated that SOD1 was significantly increased by CORM-2 in UVB-irradiated cells while its co-treatment with RES or treatment with RES alone remained constant (Figure 3A). In the absence of UVB, the combination of CORM-2 and RES upregulated the SOD1 protein expression level, suggesting that enhancement of SOD1 protein expression level could be achieved regardless of UVB irradiation. With UVB exposure, the combination of RES and CORM-2 seemed to maintain the level of SOD1. Consistent with a previous study reporting the upregulation of SOD1 protein expression under oxidative conditions [53], our results also showed that CORM-2 induced SOD1 protein expression with UVB, indicative of the important role of intracellular CO in stimulating antioxidant response after UVB treatment in HaCaT cells. Although the exact mechanism by which intracellular CO increases SOD1 independent of RES needs to be further studied, these results provide insights into the important role of intracellular CO in stimulating antioxidant enzymes under oxidative conditions. Meanwhile, mitochondrial SOD2 protein expression level was upregulated in cells treated with RES or RES and CORM-2 (Figure 3B). With UVB treatment, SOD2 protein expression level in the cytosol declined, suggesting that UVB effectively stimulated activation of SOD2 and possibly promoted its mitochondrial translocation in accordance with previously reported studies [51,52]. Although the mitochondrial SOD2 protein expression and activity after UVB irradiation need to be further examined, the current findings initially suggest that cells alleviate stress-induced damage, at least in part, through modulation of key cellular detoxifying enzymes.

## 4. Discussion

RES could exert a cytoprotective effect on skin cells exposed to UV light by modulating multiple signaling pathways [54]. Previously, we have determined that RES exerts its cytoprotective effect by upregulating HO-1 activity [55,56]. In the current study, we have investigated whether CO, one of the enzymatic products of HO-1, contributes to the cytoprotective mechanism of HO-1 against UVB challenge to the skin in human keratinocyte model, with excluding the direct antioxidant effect of bilirubin and biliverdin which are already well-documented antioxidants generated by HO-1 activity [33,57]. Considering previous evidence demonstrating that CO modulates mitochondrial function, which ultimately leads to cell homeostasis and cytoprotection [29,58], we examined whether the independent and combinatorial pre-treatment of CORM-2 and RES could exert cytoprotection against UVB-induced oxidative stress in HaCaT cells. Additionally, the effect of suppressing HO-1 activity was evaluated to determine whether CO released from CORM-2 could substitute the HO-1-mediated cytoprotection.

In the present study, keratinocytes treated with CORM-2 or RES exhibited resistance to UVB-induced oxidative stress by upregulating the protein expression of HO-1. In addition, CORM-2 reinforced the effect of RES on inducible HO-1 protein expression, which is a plausible indication of the crucial contribution of intracellular CO in regulating cellular processes by resembling the characteristics of oxidative stress. Suppression of HO-1 activity following SnPP treatment in H_2_O_2_-exposed cells significantly diminished the antioxidant activity of RES, representing a significant degeneration in stress defense system after HO-1 inhibition. Most importantly, treatment with CORM-2 after HO-1 inhibition reversed the effect of SnPP, strong evidence that CORM-2 could be an auxiliary cytoprotective agent when bilirubin production is obstructed.

The association of mitochondrial functionality with cellular detoxification and cytoprotection is a developing area of research for oxidative stress. Therefore, exploring the implications of intracellular CO-mediated adaptive signaling in UVB-irradiated keratinocytes is a valuable phase in understanding cellular behavior under oxidative conditions. In the literature, mitochondrial mass is increased in cells that encounter oxidative insults as an adaptive mechanism and stress response [59]. Although our results showed that pre-incubation with either RES or CORM-2 only maintained the mitochondrial biogenesis upon UVB treatment, we confirmed that CORM-2 slightly inhibited mitochondrial biogenesis induced by RES in the absence of UVB. This finding suggests that RES induces mitochondrial biogenesis independent of CO signaling and thus directed us to other possible mechanisms that may be involved in intracellular CO-mediated mitochondrial functionality such as respiration.

The mild inhibition in RES-induced mitochondrial respiration in its co-treatment with CORM-2 strongly support findings from other studies that CO interrupts the mitochondrial electron transport chain by competing with oxygen in its binding with heme and thus inhibiting oxygen-dependent mitochondrial enzymatic processes [58]. In addition, altering the mitochondrial respiratory chain using respective mitochondrial enzyme inhibitors reflected that CO moderately limits the mitochondrial oxygen consumption related to the key mitochondrial parameters of respiration such as ATP production, proton leak, spare respiratory capacity, maximal respiration, and non-mitochondrial respiration. Defective mitochondrial respiratory chain normally results in greater mitochondrial ROS generation and thus increases the risk of oxidative damage [60].

In addition, our data exhibited that intracellular CO upregulated the protein expressions of SOD1 and SOD2 and mildly inhibited mitochondrial respiration. It suggests that moderate inhibition of mitochondrial respiration would not spontaneously diminish the overall cellular health, but possibly participate in cell antioxidant signaling [29,61,62]. Consistently, the gradual reduction in intracellular ROS with CORM-2 treatment according to the time-dependent monitoring of intracellular ROS production involves a progressive removal of excess ROS and cellular repair through antioxidant signaling, confirming the key function of CO in maintaining redox homeostasis. This mechanism establishes the function of momentary oxidative stress in stimulating enzymatic detoxification as a part of the cellular adaptive process, in accordance with a previously described mechanism explaining the modification of mitochondrial function by CO which releases ROS signals capable of inducing cellular stress response [29].

## 5. Conclusions

**Figure 4:**
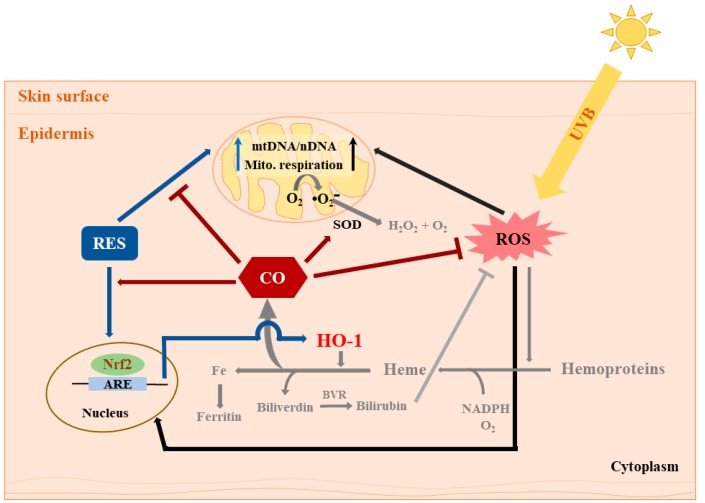
Summary of the effects of RES and CORM-2 on UVB-irradiated HaCaT cells. RES and/or CORM-2 promoted adaptive response under oxidative conditions through modulation of mitochondrial function and antioxidant enzyme signaling in HaCaT cells. Although the effect of RES on HO-1-derived CO could not be directly confirmed, this study was able to provide initial evidence regarding the participation of intracellular CO in cellular regulation against UVB-induced cytotoxicity. Pathways colored in grey indicate that the processes may possibly take place outside the cell. ARE: antioxidant response element, BVR: biliverdin reductase, CO: carbon monoxide, HO-1: heme oxygenase 1, mtDNA: mitochondrial DNA, nDNA: nuclear DNA, Nrf2: nuclear factor erythroid 2-related factor 2, RES: resveratrol, ROS: reactive oxygen species, SOD: superoxide dismutase, UVB: ultraviolet light B.

The fundamental understanding that intracellular CO mainly originates from the enzymatic activity of HO-1 depicts the possibility that RES might exert its cytoprotective effect through HO-1/CO signaling as well as HO-1/bilirubin. In summary, results presented in this study strengthen previous evidence that both RES and intracellular CO participate in cell signaling through modulation of mitochondrial function and antioxidant response leading to the elimination of intracellular ROS (Figure 4). The reduction in UVB-induced intracellular ROS also confirmed that intracellular CO could limit particularly the H_2_O_2_-mediated skin damage. Although the amount of CO attributable to the application of RES was not quantified in the current study, which limits us from defining the effect of RES on the generation of endogenous CO, it is most likely that there is a significant interaction between intracellular CO and antioxidant defense systems.

CORM-2, an excellent source of controlled amounts of CO for studying cellular mechanisms, enables the study of the biological action of intracellular CO [34]. To the best of our knowledge, the findings in the current study demonstrate for the first time the photochemoprotective effect of intracellular CO against UVB-induced damage in addition to the more well-known antioxidant function of bilirubin production resulting from HO-1 catalytic activity in the skin. While these findings strongly support earlier reports that CO could serve as a valuable target in phytochemical-based preventive healthcare and drug design against UVB-induced cytotoxicity, further investigation is recommended to evaluate the protective effect and precise action mechanism of actual intracellular CO derived from catalytic activity of HO-1. At this point, our initial hypothesis that RES regulates cellular antioxidant response via HO-1/intracellular CO signaling is not exclusively true at all circumstances as our data have illustrated that RES and CO act independently, or at least partially independently, as exemplified by the results with OCR. In conclusion, the current study underpins a crucial role of intracellular CO in modulating cellular redox homeostasis against UVB-induced oxidative stress.

## Supplementary Materials

The following are available online at https://www.mdpi.com/2076-3921/8/10/432/s1, Figure S1: Hypothetical mechanism for the cytoprotective effect of RES and CORM-2 under oxidative conditions. The current work highlights the role of intracellular CO in maintaining cellular homeostasis by upregulating the stress-inducible antioxidant enzymes to enhance cellular detoxification and ultimately maintaining redox balance in HaCaT cells. CO: carbon monoxide, CORM-2: CO-releasing molecule-2, HO-1: heme oxygenase-1, RES: resveratrol, ROS: reactive oxygen species, Figure S2: Viability of HaCaT cells treated with various concentrations of CORM-2. As concentrations above 100 µM CORM-2 were found to be cytotoxic to HaCaT cells, 100 µM was selected as the suitable treatment concentration for this study. In addition, non-cytotoxic concentrations of RES and SnPP were used consistently in this study. Cells were incubated with RES, CORM-2, or SnPP for 24 h and cytotoxicity was measured by CCK-8 assay. Results represent mean ± SD (*n* = 3). Bars not sharing common letters indicate significant differences (*p* < 0.05), as evaluated using one-way ANOVA followed by the Duncan multiple-range test. Control, DMSO; RES, resveratrol; CORM-2, CO-releasing molecule-2, SnPP, tin protoporphyrin IX., Figure S3: Viability of H_2_O_2_-exposed HaCaT cells pre-treated with RES and/or CORM-2. CORM-2 and RES increased the viability of HaCaT cells exposed to H_2_O_2_-induced cytotoxicity. Thus, RES and CORM-2 exhibit cytoprotective effect against H_2_O_2_-induced oxidative damage. Cells were pre-treated with RES, CORM-2, and/or SnPP and then exposed to H_2_O_2_. Results represent mean ± SD (*n* = 3). Bars not sharing common letters indicate significant differences (*p* < 0.05), as evaluated using one-way ANOVA followed by the Duncan multiple-range test. Control, DMSO; RES, resveratrol (30 µM); CORM-2, CO-releasing molecule-2 (100 µM), SnPP, tin protoporphyrin IX (10 µM).
