# Supplementary material for: Carbon Monoxide Partially Mediates Protective Effect of Resveratrol Against UVB-Induced Oxidative Stress in Human Keratinocytes
Source: Antioxidants (Basel). 2019 Oct 1;8(10):432. doi: 10.3390/antiox8100432 (PMC6827139; doi:10.3390/antiox8100432)
Supplement: Supplementary file 1 [file antioxidants-08-00432-s001.pdf]

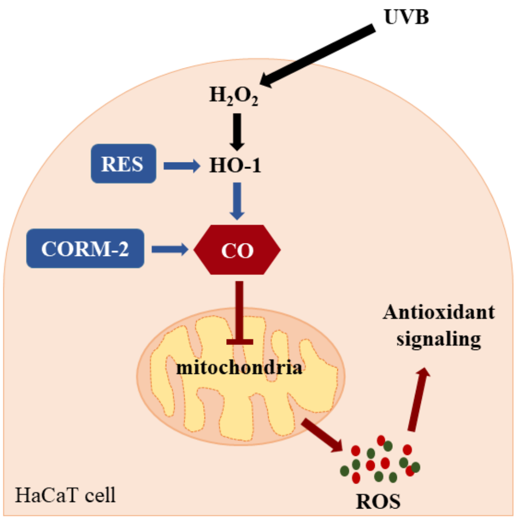

Figure 1. Hypothetical mechanism for the cytoprotective effect of RES and CORM-2 under oxidative conditions. The current work highlights the role of intracellular CO in maintaining cellular homeostasis by upregulating the stress-inducible antioxidant enzymes to enhance cellular detoxification and ultimately maintaining redox balance in HaCaT cells.

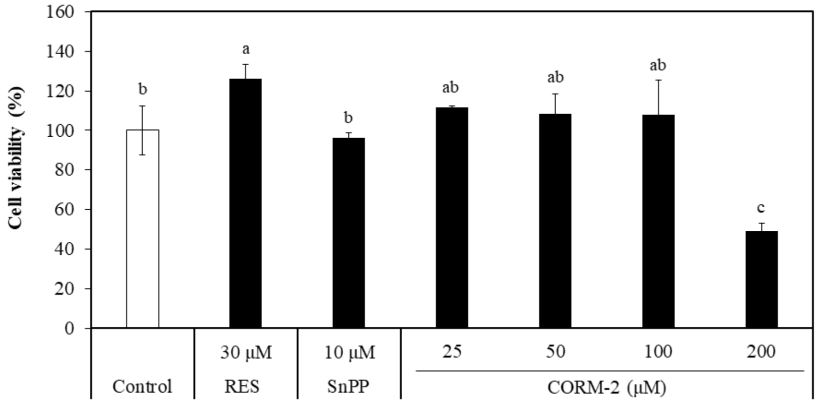

Figure 2. Viability of HaCaT cells treated with various concentrations of CORM-2. As concentrations above 100 μM CORM-2 were found to be cytotoxic to HaCaT cells, 100 μM was selected as the suitable treatment concentration for this study. In addition, non-cytotoxic concentrations of RES and SnPP were used consistently in this study. Cells were incubated with RES, CORM-2, or SnPP for 24 hrs and cytotoxicity was measured by CCK-8 assay. Results represent mean ± SD (N = 3). Bars not sharing common letters indicate significant differences ($p < 0.05$), as evaluated using one-way ANOVA followed by the Duncan multiple-range test. Control, DMSO; RES, resveratrol; CORM-2, CO-releasing molecule-2, SnPP, tin protoporphyrin IX.

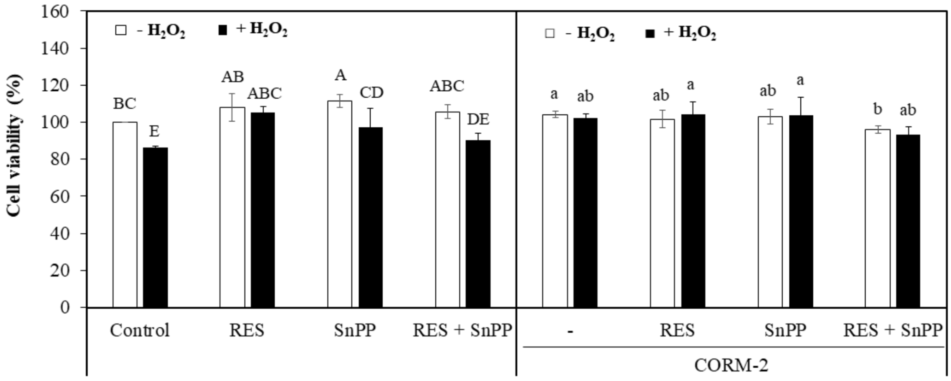

Figure 3. Viability of H_2O_2-exposed HaCaT cells pre-treated with RES and/or CORM-2. CORM-2 and RES increased the viability of HaCaT cells exposed to H_2O_2-induced cytotoxicity. Thus, RES and CORM-2 exhibit cytoprotective effect against H_2O_2-induced oxidative damage. Cells were pre-treated with RES, CORM-2, and/or SnPP and then exposed to H_2O_2. Results represent mean ± SD (n = 3). Bars not sharing common letters indicate significant differences ($p < 0.05$), as evaluated using one-way ANOVA followed by the Duncan multiple-range test. Control, DMSO; RES, resveratrol (30 μM); CORM-2, CO-releasing molecule-2 (100 μM), SnPP, tin protoporphyrin IX (10 μM).
